# Risk of Amyotrophic Lateral Sclerosis and Exposure to Particulate Matter from Vehicular Traffic: A Case-Control Study

**DOI:** 10.3390/ijerph18030973

**Published:** 2021-01-22

**Authors:** Tommaso Filippini, Jessica Mandrioli, Carlotta Malagoli, Sofia Costanzini, Andrea Cherubini, Giuseppe Maffeis, Marco Vinceti

**Affiliations:** 1 Department of Biomedical, Metabolic and Neural Sciences, CREAGEN Environmental, Genetic and Nutritional Epidemiology Research Center, University of Modena and Reggio Emilia, 41125 Modena, Italy; tommaso.filippini@unimore.it (T.F.); carlotta.malagoli@unimore.it (C.M.); 2 Neurology Unit, Department of Neuroscience, S. Agostino Estense Hospital, Azienda Ospedaliero Universitaria di Modena, 41126 Modena, Italy; mandrioli.jessica@aou.mo.it; 3 DIEF Department of Engineering “Enzo Ferrari,” University of Modena and Reggio Emilia, 41125 Modena, Italy; sofia.costanzini@unimore.it; 4 TerrAria s.r.l., 20125 Milan, Italy; a.cherubini@terraria.com (A.C.); g.maffeis@terraria.com (G.M.); 5 Department of Epidemiology, Boston University School of Public Health, Boston, MA 02118, USA

**Keywords:** amyotrophic lateral sclerosis, particulate matter, vehicular traffic, geographical information system, environmental factors, case-control study

## Abstract

(1) *Background*: Amyotrophic lateral sclerosis (ALS) is a progressive neurodegenerative disease with still unknown etiology. Some occupational and environmental risk factors have been suggested, including long-term air pollutant exposure. We carried out a pilot case-control study in order to evaluate ALS risk due to particulate matter with a diameter of ≤10 µm (PM_10_) as a proxy of vehicular traffic exposure. (2) *Methods*: We recruited ALS patients and controls referred to the Modena Neurology ALS Care Center between 1994 and 2015. Using a geographical information system, we modeled PM_10_ concentrations due to traffic emissions at the geocoded residence address at the date of case diagnosis. We computed the odds ratio (OR) and 95% confidence interval (CI) of ALS according to increasing PM_10_ exposure, using an unconditional logistic regression model adjusted for age and sex. (3) *Results*: For the 132 study participants (52 cases and 80 controls), the average of annual median and maximum PM_10_ concentrations were 5.2 and 38.6 µg/m^3^, respectively. Using fixed cutpoints at 5, 10, and 20 of the annual median PM_10_ levels, and compared with exposure <5 µg/m^3^, we found no excess ALS risk at 5–10 µg/m^3^ (OR 0.87, 95% CI 0.39–1.96), 10–20 µg/m^3^ (0.94, 95% CI 0.24–3.70), and ≥20 µg/m^3^ (0.87, 95% CI 0.05–15.01). Based on maximum PM_10_ concentrations, we found a statistically unstable excess ALS risk for subjects exposed at 10–20 µg/m^3^ (OR 4.27, 95% CI 0.69–26.51) compared with those exposed <10 µg/m^3^. However, risk decreased at 20–50 µg/m^3^ (OR 1.49, 95% CI 0.39–5.75) and ≥50 µg/m^3^ (1.16, 95% CI 0.28–4.82). ALS risk in increasing tertiles of exposure showed a similar null association, while comparison between the highest and the three lowest quartiles lumped together showed little evidence for an excess risk at PM_10_ concentrations (OR 1.13, 95% CI 0.50–2.55). After restricting the analysis to subjects with stable residence, we found substantially similar results. (4) *Conclusions*: In this pilot study, we found limited evidence of an increased ALS risk due to long-term exposure at high PM_10_ concentration, though the high statistical imprecision of the risk estimates, due to the small sample size, particularly in some exposure categories, limited our capacity to detect small increases in risk, and further larger studies are needed to assess this relation.

## 1. Introduction

Amyotrophic lateral sclerosis (ALS) is a neurodegenerative disease of the upper and lower motor neurons characterized by a fatal prognosis due to substantial respiratory or nutritional failure [1], with a median survival time from symptoms onset to death or invasive respiratory support between 24 and 50 months [2]. In Europe, ALS incidence has been increasing in recent decades, varying between 1 and 3 cases per 100,000 inhabitants [3,4,5]. Either sporadic or familial ALS forms have been described, the latter accounting for 5–10% of cases [1]. The etiology of this disease is still unknown, ALS being a complex disorder with genetic factors interacting with environmental factors in enhancing individual susceptibility [6,7,8]. Clinical and lifestyle determinants have been widely investigated, especially smoking, military service, and traumatic events [9,10,11]. Additionally, exposure to several occupational and environmental factors has been addressed, including electromagnetic fields, cyanotoxins, and chemicals such as pesticides, solvents, heavy metals, and selenium [12,13,14,15,16,17,18,19,20]. Most recently, exposure to traffic-related air pollutants has been investigated [21]. In particular, residential exposure to high levels of traffic-derived aromatic solvents was associated with increased ALS risk in the US [22]. Similarly, long-term air pollutant exposure to PM_2.5_, NO_x,,_ and NO_2_ showed increased ALS risk in highly-exposed subjects [23,24]. The Emilia-Romagna region is one of the most polluted areas of Italy and Europe, and approximately 7% of the European urban populations demonstrated exposure above the annual European limits of air pollutants [25]. Due to there still being limited evidence on the role of outdoor air pollution in ALS etiology, we aimed at investigating ALS risk due traffic-related pollutant exposure in an Italian population characterized by generally high environmental burden.

## 2. Methods

### 2.1. Study Population

Following approval from the Modena Ethics Committee (no. 85/2015), we carried out a pilot hospital-based case-control study in Modena, Northern Italy. Newly-diagnosed ALS cases were recruited at the ALS Center of Modena University Hospital, the only center specifically devoted to curing the disease in the province, from subjects who underwent lumbar puncture in the period 1994–2015 as previously described [26,27]. Only patients receiving a diagnosis of clinically definite and probable ALS were included [28]. We recruited a control population at random from all individuals admitted to the same Neurological Department in the same period, who underwent lumbar puncture because of suspected but later disconfirmed neurological disease. All subjects provided written informed consent.

### 2.2. Exposure Assessment

Using a geographical information system, we assessed individual exposure to vehicular traffic by estimating median and maximum annual levels of particulate matter <10 µm size (PM_10_), as described in detail in previous studies [29,30]. Briefly, we geocoded the residence of all study subjects at the time of diagnosis. We modeled ambient air PM_10_ levels from traffic emissions at these locations in 2006, halfway through the participant recruitment period (1994–2015) due to the limited changes over time in the number of vehicles and fuel supply [31]. We used the CAlifornia LINE version 4 (CALINE4) (Department of Transportation, Division of New Technology and Research. Sacramento, CA, USA) air quality dispersion model for roads and other linear sources. The CALINE4 model allowed us to estimate the dispersion and deposition of pollutants such as particulate matter and other contaminants at predefined spatial receptors [32]. The model was generated through the incorporation of demographic, occupational, and personal mobility data for the Modena province residents from the 2001 national census [33]. Mobility information was also validated using automatic vehicle counters in a few major roads and ad hoc surveys [34]. On the basis of estimated daily movements for Modena residents by sex, age, family structure, and occupation, we computed a matrix of vehicle movements for each road in the province [35]. In addition, we validated the model within the study area by comparing modeled PM_10_ levels with measured concentration at air monitoring stations [34].

### 2.3. Statistical Analysis

We used crude and adjusted multivariate unconditional logistic regression models to estimate odds ratios (ORs) and 95% confidence intervals (CIs) of ALS associated with PM_10_ increasing exposures using annual median and maximum PM_10_ levels. For median levels, we used the values established in the “WHO air quality guidelines” as upper cutpoints: these are 20 μg/m^3^ annual mean PM_10_ levels, and 50 μg/m^3^ for 24 h maximum mean PM_10_ levels [36]. Accordingly, we calculated ALS risk for median PM_10_ levels of 5–10 µg/m^3^, 10–20 µg/m^3^, and ≥20 µg/m^3^, using the <5 µg/m^3^ category as a reference. Similarly, we computed ALS risk for the maximum PM_10_ level categories of 10–20 µg/m^3^, 20–50 µg/m^3,^ and ≥50 µg/m^3^, using the <10 µg/m^3^ category as a reference. In addition, we calculated ALS risk according to increasing tertiles of PM_10_ exposure based on the distribution of the control population, using the lowest tertile as a reference. We also used compared ALS risk for subjects experiencing PM_10_ concentrations above the highest quartile, based on the distribution of controls, compared with the three lowest quartiles lumped together. Finally, we modeled the relation between PM_10_ exposure and ALS risk using a restricted cubic spline model with three knots at either fixed percentiles (10th, 50th, and 90th) or categories (5, 10, and 20 µg/m^3^ for annual median PM_10_ levels, and 10, 20, and 50 µg/m^3^ for annual maximum PM_10_ levels).

We ran all analyses in all subjects and only in those who did not change their residence in the last five years before ALS diagnosis. We included sex and age as potential confounders and effect-modifiers in a multivariable model. We further adjusted for residential passive exposure to pesticides as assessed using proximity to agricultural land use [37,38,39] or inorganic selenium species [26]. We used ‘logit’, ‘mkspline’, and ‘xblc’ routines of the Stata 16.1 (Stata Corp., College Station, TX, USA) for all statistical analyses.

## 3. Results

Of 145 eligible subjects, we eventually recruited 132 residents from the Modena province. These included 52 ALS cases and 80 controls (Table 1), of whom 70 men (cases/controls: 31/39) and 62 women (cases/controls: 21/41), with a mean age higher in cases (58.2 years) compared to controls (52.8 years). Subjects excluded due to residence in another province had similar sex and age distribution compared with the included participants (Supplementary Table S1).

Annual median and maximum PM_10_ levels showed similar values in cases and controls, with an overall mean of 5.2 µg/m^3^ and 38.6 µg/m^3^, respectively (Table 1). Using fixed cutpoints at 5, 10, and 20 µg/m^3^ of annual median PM_10_ levels, compared to exposure <5 µg/m^3^ (Table 2), we found a slightly increased ASL risk in the highest category only (OR = 1.50, 95% CI 0.09–24.92). However, after adjusting for sex and age, such increased ALS risk did not persist at any exposure level (Table 2). When using annual maximum PM_10_ levels, we found a statistically imprecise excess ALS risk for the 10–20 µg/m^3^ exposure category compared with <10 µg/m^3^ in both the crude (OR = 3.00, 95% CI 0.52–17.16) and adjusted (OR = 4.27, 95% CI 0.69–26.51) model (Table 2). However, such excess risk decreased with further increase in exposure, i.e., at 20–50 µg/m^3^ and above 50 µg/m^3^.

After excluding subjects (*N* = 9) who moved their residence within five years of ALS diagnosis, we found similar results for annual maximum PM_10_ exposure, with the highest ALS risk in the intermediate exposure categories in both the crude and adjusted model (Table 3). Conversely, in relation to median PM_10_ exposure, we found a dose-dependent but imprecise ALS risk increase in the crude model (ORs 1.17, 1.36, and 2.05) after adjusting for age and sex, however, such effect disappeared with the highest risk in the 10–20 µg/m^3^ exposure category (OR 1.35, 95% CI 0.34–5.43).

In the analysis using tertile distribution, we did not find an indication of an increased ALS risk at high exposure levels neither for median or maximum PM_10_ concentrations (Table 4), although the median exposure levels were much lower since the majority of subjects experienced PM_10_ concentrations below 7 µg/m^3^ and 50 µg/m^3^, respectively, thus influencing the reference group exposure compared with the analysis using fixed categories.

After exclusion of subjects without stable residence in the last 5 years, results were substantially comparable, although there was some indication of an increased risk in the crude analysis for annual median PM_10_ levels. In such an analysis, ORs were 1.20 (95% CI 0.47–3.05) and 1.28 (95% 0.51–3.23) in the second and third tertile, respectively, although multivariate analysis showed a slightly increased but statistically imprecise ALS risk in the highest tertile only (OR 1.07, 95% CI 0.41–1.79) (Table 5).

Analysis comparing the highest with the three lowest quartiles lumped together showed an increased ALS risk for subjects in the highest category for annual median PM_10_ levels, with OR of 1.13 (95% CI 0.50–2.55) in the adjusted model, while annual maximum PM_10_ levels did not demonstrate such increased risk (Table 6). The exclusion of subjects without stable residence confirmed and further strengthened these results (Table 7).

Further adjusted for agricultural land use or inorganic selenium levels in cerebrospinal fluid showed substantially comparable results (Supplementary Tables S2–S7). Only an adjustment for selenium led to a slight enhancement of ALS increased risk in both analyses using fixed cutpoints (Supplementary Tables S2 and S3) and percentile distribution (Supplementary Tables S4–S7), although these latter analyses due to availability of inorganic selenium assessment were performed in a subset of participants, i.e., 94 subjects (34 cases and 60 controls) in the main analysis, and 87 subjects (27 cases and 60 controls) in the analysis excluding those without a stable residence.

**Figure 1:**
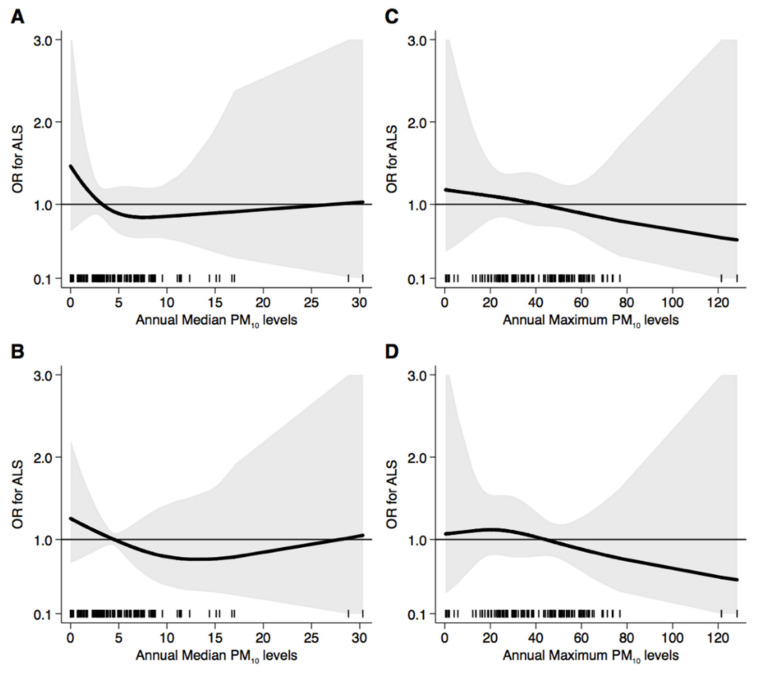
Spline regression analysis of amyotrophic lateral sclerosis (ALS) risk for increasing particulate matter (PM_10_) exposure for both annual median and maximum levels using 10th, 50th, and 90th percentile distribution (**A**,**C**) or fixed categories ((**B**): 5, 10 and 20 µg/m^3^; (**D**): 10, 20 and 50 µg/m^3^) as cutpoints. The black line indicates the odds ratio for ALS risk; the gray area indicates 95% confidence limits; the reference line is at 1.0; black spikes indicate the distribution of participant PM_10_ levels.

**Figure 2:**
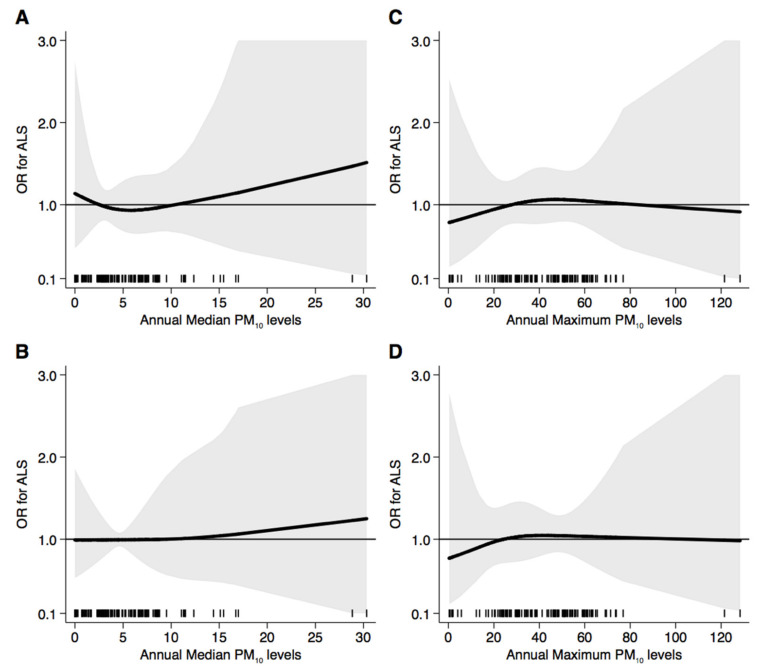
Spline regression analysis of amyotrophic lateral sclerosis (ALS) risk for increasing particulate matter (PM_10_) exposure for both annual median and maximum levels using 10th, 50th, and 90th percentile distribution (**A**,**C**) or fixed categories ((**B**): 5, 10, and 20 µg/m^3^; (**D**): 10, 20, and 50 µg/m^3^) as cutpoints. The black line indicates the odds ratio for ALS risk; the gray area indicates 95% confidence limits; the reference line is at 1.0; black spikes indicate the distribution of participant PM_10_ level. Sensitivity analysis of subjects with stable residence within five years of diagnosis.

Figure 1 presents the results of the spline regression analysis of ALS risk, showing no substantial change in disease risk at every exposure level for both annual median and maximum PM_10_ exposure. Moreover, no relevant difference emerged using fixed percentiles or fixed categories to identify the knots of the regression model. After exclusion of subjects who moved their residence within five years of diagnosis (Figure 2), we found substantially similar results, except for a statistically highly imprecise but increased risk above 20 µg/m^3^ of annual median PM_10_ exposure.

Further adjustment for agricultural land use did not substantially change the shape of the spline analysis in both overall subjects and in those with stable residence (Supplementary Figures S1 and S2). Conversely, adjustment for inorganic selenium levels showed an increased ALS risk in the analysis using annual median PM_10_ exposure, although both analyses in overall subjects (Supplementary Figure S3) and especially in the one excluding subjects without stable residence (Supplementary Figure S4) were characterized with very high statistical imprecision, hampering the interpretation of the risk estimates.

## 4. Discussion

In this pilot study, we found little indication of an association between outdoor pollution, as assessed through PM_10_ levels from vehicular traffic, and ALS risk, except for a statistically imprecise excess risk based on annual median exposure levels above 20 µg/m^3^. In recent years, several public actions have been taken to decrease the environmental burden of outdoor air pollutants, e.g., a new vehicle pollution abatement system to cut emissions and economic benefits for vehicle renewal [40]. In particular, the Emilia-Romagna region since 2013 implemented a plan to cut pollutant emissions, especially PM_10_ concentrations [41]. Despite such measures, environmental emissions are still of concern, and exposure to outdoor air pollution has been associated with several adverse health effects on humans, including increased incidence or mortality for cancer as well as infectious, respiratory, and cardiovascular diseases [42,43,44,45,46]. In particular, a link has also been suggested between high levels of air pollutants and neurodegenerative disorders, e.g., Parkinson’s disease, multiple sclerosis, and Alzheimer’s dementia [47,48,49,50]. Previous studies assessing ALS risk in relation to long-term air pollution exposure suggested an increase in risk due to high levels of both PM_2.5_ and NO_x_ in the Netherlands [23]. Similarly, a dose-response relation between increasing exposure to NO_2_ and ALS risk has been reported in a study carried out in Catalonia, Spain [24]. Occupational studies consistently found an increased ALS risk in categories characterized by generally high exposure to traffic exhausts [51], including truck and bus drivers, mechanics and repairers, or auto repair, service and gas station attendants [52,53,54].

Toxicological and animal studies provide some biological plausibility for an association between air pollutants and neurodegeneration. For instance, they indicate that airborne pollutants, especially ultrafine particles, are able to cross the blood-brain barrier and enter the brain through the olfactory nerve after deposition on the olfactory mucosa [55]. Moreover, ambient particulate matter and other airborne pollutants have been associated with markers of neuroinflammation of various regions of the central nervous system, including elevated oxidative stress, cytokine expression, increased lipid peroxidation, and microglia activation [56,57], especially in the hippocampus and the olfactory bulb [58,59]. In an animal model, similarly, chronic exposure to PM resulted in a dose-dependent increase in pure cortical neuronal loss and selective neuronal loss especially of the motor cortex, primary somatosensory cortex, and piriform cortex [60]. In addition, traffic-related pollution may contain elements characterized by neurotoxic effects, especially heavy metals such as lead, cadmium, mercury, and selenium already associated with ALS risk [61,62,63,64,65,66]. A neuroimaging study showed that long-term exposure to high ambient air pollution may lead to cortical thinning and reduced subcortical volume in adults [67].

A few limitations of our study should be pointed out. Exposure assessment relied on PM_10_ concentrations only, and not on other outdoor pollutants such as benzene. Nonetheless, in a previous study we carried out assessing both PM_10_ and benzene emissions from vehicular traffic in two municipalities from the same area (i.e., Modena and Reggio Emilia), we found a correlation between the two pollutants, with a Pearson’s correlation coefficient of 0.53 [34]. In addition, we did not evaluate other pollutants such as nitrogen dioxide, ozone, and carbon monoxide, although their correlation with PM_10_ may not have been very high due to the relevance of domestic sources (i.e., energy production, heat boilers) in these emissions [25,68], while our study specifically focused on vehicular traffic air emissions. Secondly, the small sample size has affected the precision of the risk estimates, especially for the low number of exposed subjects in some exposure categories based on fixed cutpoints, hampering their interpretation. As regards to the reference population, controls were recruited from subjects referred to Modena Hospital Neurological Department, although subsequently discharged without a diagnosis of neurological disease. In addition, due to the nonexperimental study design and the limited availability of personal information except for sex and age, we cannot entirely rule out the presence of residual confounding. Moreover, we relied on a geographical information system (GIS) for exposure assessment. This methodology avoided recall bias but was still open to other possible sources of bias, such as changes in air pollution at the participants’ residence over time. In particular, we cannot entirely rule out the possible occurrence of an amount of exposure misclassification, especially due to the use of the residential address not taking into account exposure occurred during occupational activities and place of work. During the study period, however, only slight changes occurred in the Modena municipality, where most participants were residents, in terms of the number of vehicles (113,648 in 1994, 117,034 in 2006, and 116,693 in 2015) and adult population (from 151,170 in 1994, 152,372 in 2006, and 154,718 in 2015) [31]. In addition, only a modest increase was observed in the rate of vehicles/1000 adult population at a provincial level, from 721,302 in 1994 to 759,719 in 2006 and 773,713 in 2015 [31]. As regards to fuel supply, a small increase in diesel use has been registered in Italy in most recent years [69]. However, PM_10_ concentration did not show such a comparable trend, demonstrating instead a slight decrease due to the implementation of new devices to cut emissions such as particulate filters [70]. Additionally, the age and sex distribution of excluded subjects were similar to those of study participants, thus reducing the likelihood of selection bias. Finally, we used the threshold values suggested by the WHO as cutpoints for risk analysis, for which evidence of adverse health effects at these exposure levels in urban populations in both developed and developing countries have already been reported [36], thus allowing for easier comparison across studies and in meta-analyses. 

## 5. Conclusions

Overall, we found limited evidence of an excess in ALS risk due to long-term exposure at high PM_10_ concentrations, although an increased risk at annual median exposure levels above 20 µg/m^3^ cannot be excluded. However, our capacity to detect small increases in risk was limited by the statistical imprecision of the risk estimates due to the small sample size, particularly in some exposure categories. Therefore, we acknowledge that further and larger studies are needed to definitively assess the association between traffic-related air pollution and amyotrophic lateral sclerosis.

## Figures and Tables

**Table 1:** Characteristics of the study population. Number (%) of subjects reported.

Characteristics	Cases	Controls	All Subjects
All subjects	52 (100)	80 (100)	132 (100)
Sex			
Men	31 (59.6)	39 (48.7)	70 (53.0)
Women	21 (40.4)	41 (51.3)	62 (47.0)
Age ^1^	58.2 (12.6)	52.8 (15.4)	54.9 (14.5)
<65 years	35 (67.3)	59 (73.7)	94 (71.2)
≥65 years	17 (32.7)	21 (26.3)	38 (28.8)
Annual median PM_10_ levels ^1^	5.1 (5.0)	5.3 (4.6)	5.2 (4.8)
Annual maximum PM_10_ levels ^1^	37.2 (22.5)	39.4 (21.3)	38.6 (21.7)

^1^: Mean (standard deviation).

**Table 2:** Risk of amyotrophic lateral sclerosis according to particulate matter (PM_10_) exposure using fixed cutpoints.

Exposure	Cases/Controls	OR ^a^	(95% CI)	OR ^b^	(95% CI)
Annual median PM_10_ levels					
<5 µg/m^3^	30/45	1.00	-	1.00	-
5–10 µg/m^3^	17/28	0.91	(0.43–1.95)	0.87	(0.39–1.96)
10–20 µg/m^3^	4/6	1.00	(0.26–3.85)	0.94	(0.24–13.70)
≥20 µg/m^3^	1/1	1.50	(0.09–24.92)	0.87	(0.05–15.01)
Annual maximum PM_10_ levels					
<10 µg/m^3^	4/8	1.00	-	1.00	-
10–20 µg/m^3^	6/4	3.00	(0.52–17.16)	4.27	(0.69–26.51)
20–50 µg/m^3^	28/40	1.40	(0.38–5.10)	1.49	(0.39–5.75)
≥50 µg/m^3^	14/28	1.00	(0.26–3.90)	1.16	(0.28–4.82)

^a^: Crude model; ^b^: Model adjusted for sex and age. OR: odds ratio; CI: confidence interval.

**Table 3:** Risk of amyotrophic lateral sclerosis according to particulate matter (PM_10_) exposure in subjects with stable residence within five years of diagnosis using fixed cutpoints.

Exposure	Cases/Controls	OR ^a^	(95% CI)	OR ^b^	(95% CI)
Annual median PM_10_ levels					
<5 µg/m^3^	22/45	1.00	-	1.00	-
5–10 µg/m^3^	16/28	1.17	(0.53–2.60)	1.02	(0.43–2.42)
10–20 µg/m^3^	4/6	1.36	(0.35–5.33)	1.35	(0.34–5.43)
≥20 µg/m^3^	1/1	2.05	(0.12–34.26)	1.16	(0.07–20.11)
Annual maximum PM_10_ levels					
<10 µg/m^3^	3/8	1.00	-	1.00	-
10–20 µg/m^3^	2/4	1.33	(0.15–11.50)	2.19	(0.23–20.91)
20–50 µg/m^3^	24/40	1.60	(0.39–6.62)	1.64	(0.37–7.27)
≥50 µg/m^3^	14/28	1.33	(0.231–5.82)	1.53	(0.32–7.23)

^a^: Crude model; ^b^: Model adjusted for sex and age. OR: odds ratio; CI: confidence interval.

**Table 4:** Risk of amyotrophic lateral sclerosis according to particulate matter (PM_10_) exposure using tertile distribution.

Exposure Categories	Median Value (µg/m^3^)	Cases/ Controls	OR ^a^	(95% CI)	OR ^b^	(95% CI)
Annual median PM_10_ levels						
T1 < 3.0 µg/m^3^	1.4	19/26	1.00	-	1.00	-
T2 ≥ 3.0–<6.6 µg/m^3^	4.2	17/27	0.86	(0.37–2.01)	0.66	(0.28–1.68)
T3 ≥ 6.6 µg/m^3^	8.4	16/27	0.81	(0.34–1.91)	0.73	(0.30–1.79)
Annual maximum PM_10_ levels						
T1 < 30.0 µg/m^3^	20.5	22/26	1.00	-	1.00	-
T2 ≥ 30.0–<50.4 µg/m^3^	38.3	16/27	0.70	(0.30–1.62)	0.73	(0.30–1.74)
T3 ≥ 50.4 µg/m^3^	59.5	14/27	0.61	(0.26–1.45)	0.65	(0.26–1.58)

^a^: Crude model; ^b^: Model adjusted for sex and age. OR: odds ratio; CI: confidence interval.

**Table 5:** Risk of amyotrophic lateral sclerosis according to particulate matter (PM_10_) exposure in subjects with stable residence within five years of diagnosis using tertile distribution.

Exposure	Median Value (µg/m^3^)	Cases/ Controls	OR ^a^	(95% CI)	OR ^b^	(95% CI)
Annual median PM_10_ levels						
T1 < 3.0 µg/m^3^	1.4	12/26	1.00	-	1.00	-
T2 ≥ 3.0–<6.6 µg/m^3^	4.2	15/27	1.20	(0.47–3.05)	0.84	(0.31–2.29)
T3 ≥ 6.6 µg/m^3^	8.4	16/27	1.28	(0.51–3.23)	1.07	(0.41–2.83)
Annual maximum PM_10_ levels						
T1 < 30.0 µg/m^3^	22.2	14/26	1.00	-	1.00	-
T2 ≥ 30.0–<50.4 µg/m^3^	38.2	15/27	1.03	(0.42–2.55)	1.07	(0.41–2.79)
T3 ≥ 50.4 µg/m^3^	59.5	14/27	0.96	(0.39–2.41)	1.04	(0.40–2.73)

^a^: Crude model; ^b^: Model adjusted for sex and age. OR: odds ratio; CI: confidence interval.

**Table 6:** Risk of amyotrophic lateral sclerosis according to particulate matter (PM_10_) exposure comparing the highest and the three lowest quartiles lumped together.

Exposure	Cases/ Controls	OR ^a^	(95% CI)	OR ^b^	(95% CI)
Annual median PM_10_ levels					
Q1–Q3 < 7.3 µg/m^3^	38/60	1.00	-	1.00	-
Q4 ≥ 7.3 µg/m^3^	14/20	1.11	(0.50–2.45)	1.13	(0.50–2.55)
Annual maximum PM_10_ levels					
Q1–Q3 < 55.0 µg/m^3^	45/60	1.00	-	1.00	-
Q4 ≥ 55.0 µg/m^3^	7/20	0.47	(0.18–1.20)	0.45	(0.17–1.17)

^a^: Crude model; ^b^: Model adjusted for sex and age. OR: odds ratio; CI: confidence interval.

**Table 7:** Risk of amyotrophic lateral sclerosis according to particulate matter (PM_10_) exposure in subjects with stable residence within five years of diagnosis comparing the highest and the three lowest quartiles lumped together.

Exposure	Cases/ Controls	OR ^a^	(95% CI)	OR ^b^	(95% CI)
Annual median PM_10_ levels					
Q1–Q3 < 7.3 µg/m^3^	29/60	1.00	-	1.00	-
Q4 ≥ 7.3 µg/m^3^	14/20	1.45	(0.64–3.27)	1.51	(0.65–3.49)
Annual maximum PM_10_ levels					
Q1–Q3 < 55.0 µg/m^3^	36/60	1.00	-	1.00	-
Q4 ≥ 55.0 µg/m^3^	7/20	0.58	(0.22–1.52)	0.56	(0.21–1.49)

^a^: Crude model; ^b^: Model adjusted for sex and age. OR: odds ratio; CI: confidence interval.

## Data Availability

The data presented in this study may be available on reasonable request from the corresponding author. The data are not publicly available due to confidential and ethical issues.

## Supplementary Materials

The following are available online at https://www.mdpi.com/1660-4601/18/3/973/s1, Table S1. Characteristics of the excluded study population. Number (%) of subjects reported. Table S2. Risk of amyotrophic lateral sclerosis according to particulate matter (PM_10_) exposure using fixed cutpoints with further adjustment for agricultural land use or inorganic selenium. Table S3. Risk of amyotrophic lateral sclerosis according to particulate matter (PM_10_) exposure using fixed cutpoints in subjects with stable residence within five years of diagnosis with further adjustment for agricultural land use or inorganic selenium. Table S4. Risk of amyotrophic lateral sclerosis according to particulate matter (PM_10_) exposure using tertile distribution with further adjustment for agricultural land use or inorganic selenium. Table S5. Risk of amyotrophic lateral sclerosis according to particulate matter (PM_10_) exposure using tertile distribution in subjects keeping a stable residence in the five years before diagnosis with further adjustment for agricultural land use or inorganic selenium. Table S6. Risk of amyotrophic lateral sclerosis according to particulate matter (PM_10_) exposure comparing the highest and the three lowest quartiles lumped together with further adjustment for agricultural land use or inorganic selenium. Table S7. Risk of amyotrophic lateral sclerosis according to particulate matter (PM_10_) exposure comparing the highest and the three lowest quartiles lumped together in subjects keeping a stable residence in the five years before diagnosis with further adjustment for agricultural land use or inorganic selenium. Figure S1. Spline regression analysis of amyotrophic lateral sclerosis (ALS) risk for increasing particulate matter (PM_10_) exposure for both annual median and maximum levels using 10th, 50th, and 90th percentile distribution (A,C) or fixed categories (B: 5, 10, and 20 µg/m^3^; D: 10, 20, and 50 µg/m^3^) as cutpoints with further adjustment for agricultural land use. The black line indicates the odds ratio for ALS risk; the gray area indicates 95% confidence limits; the reference line is at 1.0; black spikes indicate the distribution of participant PM_10_ levels. Figure S2. Spline regression analysis of amyotrophic lateral sclerosis (ALS) risk for increasing particulate matter (PM_10_) exposure for both annual median and maximum levels using 10th, 50th, and 90th percentile distribution (A,C) or fixed categories (B: 5, 10, and 20 µg/m^3^; D: 10, 20 and 50 µg/m^3^) as cutpoints with further adjustment for agricultural land use. The black line indicates the odds ratio for ALS risk; the gray area indicates 95% confidence limits; the reference line is at 1.0; black spikes indicate the distribution of participant PM_10_ levels. Figure S3. Spline regression analysis of amyotrophic lateral sclerosis (ALS) risk for increasing particulate matter (PM_10_) exposure for both annual median and maximum levels using 10th, 50th, and 90th percentile distribution (A,C) or fixed categories (B: 5, 10, and 20 µg/m^3^; D: 10, 20, and 50 µg/m^3^) as cutpoints with further adjustment for inorganic selenium levels in cerebrospinal fluid. The black line indicates the odds ratio for ALS risk; the gray area indicates 95% confidence limits; the reference line is at 1.0; black spikes indicate the distribution of participant PM_10_ levels. Figure S4. Spline regression analysis of amyotrophic lateral sclerosis (ALS) risk for increasing particulate matter (PM_10_) exposure for both annual median and maximum levels using 10th, 50th, and 90th percentile distribution (A,C) or fixed categories (B: 5, 10, and 20 µg/m^3^; D: 10, 20, and 50 µg/m^3^) as cutpoints with further adjustment for inorganic selenium levels in cerebrospinal fluid. The black line indicates the odds ratio for ALS risk; the gray area indicates 95% confidence limits; the reference line is at 1.0; black spikes indicate the distribution of participant PM_10_ levels.
