# Supplementary material for: Risk of Amyotrophic Lateral Sclerosis and Exposure to Particulate Matter from Vehicular Traffic: A Case-Control Study
Source: Int J Environ Res Public Health. 2021 Jan 22;18(3):973. doi: 10.3390/ijerph18030973 (PMC7908475; doi:10.3390/ijerph18030973)
Supplement: Supplementary file 1 [file ijerph-18-00973-s001.pdf]

International Journal of Environmental Research and Public Health

MDPI

SUPPLEMENTAL MATERIAL

Table S1. Characteristics of the excluded study population. Number (%) of subjects reported.

Characteristics	Cases	Controls	All subjects
All subjects	7 (100)	6 (100)	13 (100)
Sex			
Men	4 (57.1)	5 (83.3)	9 (69.2)
Women	3 (42.9)	1 (16.7)	4 (30.8)
Age [1]	54.5 (3.7)	51.7 (17.0)	53.2 (14.7)
<65 years	5 (71.4)	5 (83.3)	10 (76.9)
≥65 years	2 (28.6)	1 (16.7)	3 (23.1)
Annual median PM_{10} levels [1]	-	-	-
Annual maximum PM_{10} levels [1]	-	-	-

[1] Mean (standard deviation).

Table S2. Risk of amyotrophic lateral sclerosis according to particulate matter (PM_{10}) exposure using fixed cutpoints with further adjustment for agricultural land use or inorganic selenium

Exposure	Cases/ Controls	OR [a]	(95% CI)	Cases/ Controls	OR [b]	(95% CI)
Annual median PM_{10} levels						
<5 μg/m³	30/45	1.00	-	18/35	1.00	-
5-10 μg/m³	17/28	0.84	(0.36 - 1.94)	13/19	1.07	(0.39 - 2.93)
10-20 μg/m³	4/6	0.91	(0.23 - 3.63)	3/6	0.82	(0.17 - 4.02)
≥20 μg/m³	1/1	0.83	(0.05 - 14.55)	0/0	-	-
Annual maximum PM_{10} levels						
<10 μg/m³	4/8	1.00	-	3/6	1.00	-
10-20 μg/m³	6/4	4.39	(0.71 - 27.14)	4/2	7.59	(0.73 - 79.24)
20-50 μg/m³	28/40	1.29	(0.32 - 5.27)	17/32	1.24	(0.24 - 6.33)
≥50 μg/m³	14/28	0.97	(0.22 - 4.35)	10/20	1.17	(0.21 - 6.57)

[a] Model adjusted for sex, age, and agricultural land use [b] Model adjusted for sex, age and inorganic selenium levels in cerebrospinal fluids. OR, odds ratio; CI, confidence interval.

Table S3. Risk of amyotrophic lateral sclerosis according to particulate matter (PM_{10}) exposure using fixed cutpoints in subjects with stable residence within five years of diagnosis with further adjustment for agricultural land use or inorganic selenium.

Exposure	Cases/ Controls	OR [a]	(95% CI)	Cases/ Controls	OR [b]	(95% CI)
Annual median PM_{10} levels						
<5 μg/m³	22/45	1.00	-	12/35	1.00	-
5-10 μg/m³	16/28	1.01	(0.42 - 2.46)	12/19	1.21	(0.41 - 3.63)
10-20 μg/m³	4/6	1.34	(0.33 - 5.45)	3/6	1.32	(0.26 - 6.75)
≥20 μg/m³	1/1	1.14	(0.06 - 20.10)	0/0	-	-
Annual maximum PM_{10} levels						
<10 μg/m³	3/8	1.00	-	2/6	1.00	-
10-20 μg/m³	2/4	2.19	(0.23 - 20.97)	1/2	4.78	(0.22 - 105.57)
20-50 μg/m³	24/40	1.61	(0.34 - 7.73)	14/32	1.39	(0.21 - 9.24)
≥50 μg/m³	14/28	1.50	(0.28 - 7.94)	10/20	1.67	(0.23 - 11.89)

[a] Model adjusted for sex, age, and agricultural land use [b] Model adjusted for sex, age and inorganic selenium levels in cerebrospinal fluids. OR, odds ratio; CI, confidence interval.

Table S4. Risk of amyotrophic lateral sclerosis according to particulate matter (PM_{10}) exposure using tertile distribution with further adjustment for agricultural land use or inorganic selenium.

Exposure categories	Median value (μg/m³)	Cases/ Controls	OR [a]	(95% CI)	Median value (μg/m³)	Cases/ Controls	OR [b]	(95% CI)
Annual median PM_{10} levels								
T1 <3.0 μg/m³	1.4	19/26	1.00	-	1.4	12/20	1.00	-
T2 ≥3.0-<6.6 μg/m³	4.2	17/27	0.63	(0.25 - 1.62)	4.3	11/22	0.57	(0.19 - 1.75)
T3 ≥6.6 μg/m³	8.4	16/27	0.67	(0.26 - 1.73)	8.4	11/18	0.79	(0.25 - 2.50)
Annual maximum PM_{10} levels								
T1 <30.0 μg/m³	20.5	22/26	1.00	-	20.2	14/17	1.00	-
T2 ≥30.0-<50.4 μg/m³	38.3	16/27	0.71	(0.29 - 1.70)	38.2	10/24	0.40	(0.13 - 1.24)
T3 ≥50.4 μg/m³	59.5	14/27	0.61	(0.24 - 1.54)	58.8	10/19	0.52	(0.16 - 1.64)

[a] Model adjusted for sex, age, and agricultural land use [b] Model adjusted for sex, age and inorganic selenium levels in cerebrospinal fluids. OR, odds ratio; CI, confidence interval.

Table S5. Risk of amyotrophic lateral sclerosis according to particulate matter (PM_{10}) exposure using tertile distribution in subjects keeping a stable residence in the five years before diagnosis with further adjustment for agricultural land use or inorganic selenium.

Exposure categories	Median value (μg/m³)	Cases/ Controls	OR [a]	(95% CI)	Median value (μg/m³)	Cases/ Controls	OR [b]	(95% CI)
Annual median PM_{10} levels								
T1 <3.0 μg/m³	1.4	12/26	1.00	-	1.4	7/20	1.00	-
T2 ≥3.0-<6.6 μg/m³	4.2	15/27	0.81	(0.28 - 2.33)	4.3	9/22	0.63	(0.17 - 2.33)
T3 ≥6.6 μg/m³	8.4	16/27	1.04	(0.37 - 2.90)	8.4	11/18	1.16	(0.33 - 4.13)
Annual maximum PM_{10} levels								
T1 <30.0 μg/m³	22.2	14/26	1.00	-	20.6	8/17	1.00	-
T2 ≥30.0-<50.4 μg/m³	38.2	15/27	1.06	(0.41 - 2.79)	38.1	9/24	0.55	(0.15 - 1.97)
T3 ≥50.4 μg/m³	59.5	14/27	1.02	(0.38 - 2.78)	58.9	10/19	0.85	(0.24 - 2.96)

[a] Model adjusted for sex, age, and agricultural land use [b] Model adjusted for sex, age and inorganic selenium levels in cerebrospinal fluids. OR, odds ratio; CI, confidence interval.

Table S6. Risk of amyotrophic lateral sclerosis according to particulate matter (PM_{10}) exposure comparing the highest and the three lowest quartiles lumped together with further adjustment for agricultural land use or inorganic selenium.

Exposure	Cases/ Controls	OR [a]	(95% CI)	Cases/ Controls	OR [b]	(95% CI)
Annual median PM_{10} levels						
Q1-Q3 <7.3 μg/m³	38/60	1.00	-	24/46	1.00	-
Q4 ≥7.3 μg/m³	14/20	1.11	(0.49 - 2.55)	10/14	1.44	(0.42 - 3.99)
Annual maximum PM_{10} levels						
Q1-Q3 <55.0 μg/m³	45/60	1.00	-	29/46	1.00	-
Q4 ≥55.0 μg/m³	7/20	0.43	(0.16 - 1.13)	4/14	0.49	(0.15 - 1.58)

[a] Model adjusted for sex, age, and agricultural land use [b] Model adjusted for sex, age and inorganic selenium levels in cerebrospinal fluids. OR, odds ratio; CI, confidence interval.

Table S7. Risk of amyotrophic lateral sclerosis according to particulate matter (PM_{10}) exposure comparing the highest and the three lowest quartiles lumped together in subjects keeping a stable residence in the five years before diagnosis with further adjustment for agricultural land use or inorganic selenium.

Exposure	Cases/ Controls	OR [a]	(95% CI)	Cases/ Controls	OR [b]	(95% CI)
Annual median PM_{10} levels						
Q1-Q3 <7.3 μg/m³	29/60	1.00	-	17/46	1.00	-
Q4 ≥7.3 μg/m³	14/20	1.51	(0.64 - 3.55)	10/14	2.06	(0.71 - 6.01)
Annual maximum PM_{10} levels						
Q1-Q3 <55.0 μg/m³	36/60	1.00	-	22/46	1.00	-
Q4 ≥55.0 μg/m³	7/20	0.54	(0.20 - 1.46)	5/14	0.65	(0.19 - 2.17)

[a] Model adjusted for sex, age, and agricultural land use [b] Model adjusted for sex, age and inorganic selenium levels in cerebrospinal fluids. OR, odds ratio; CI, confidence interval.

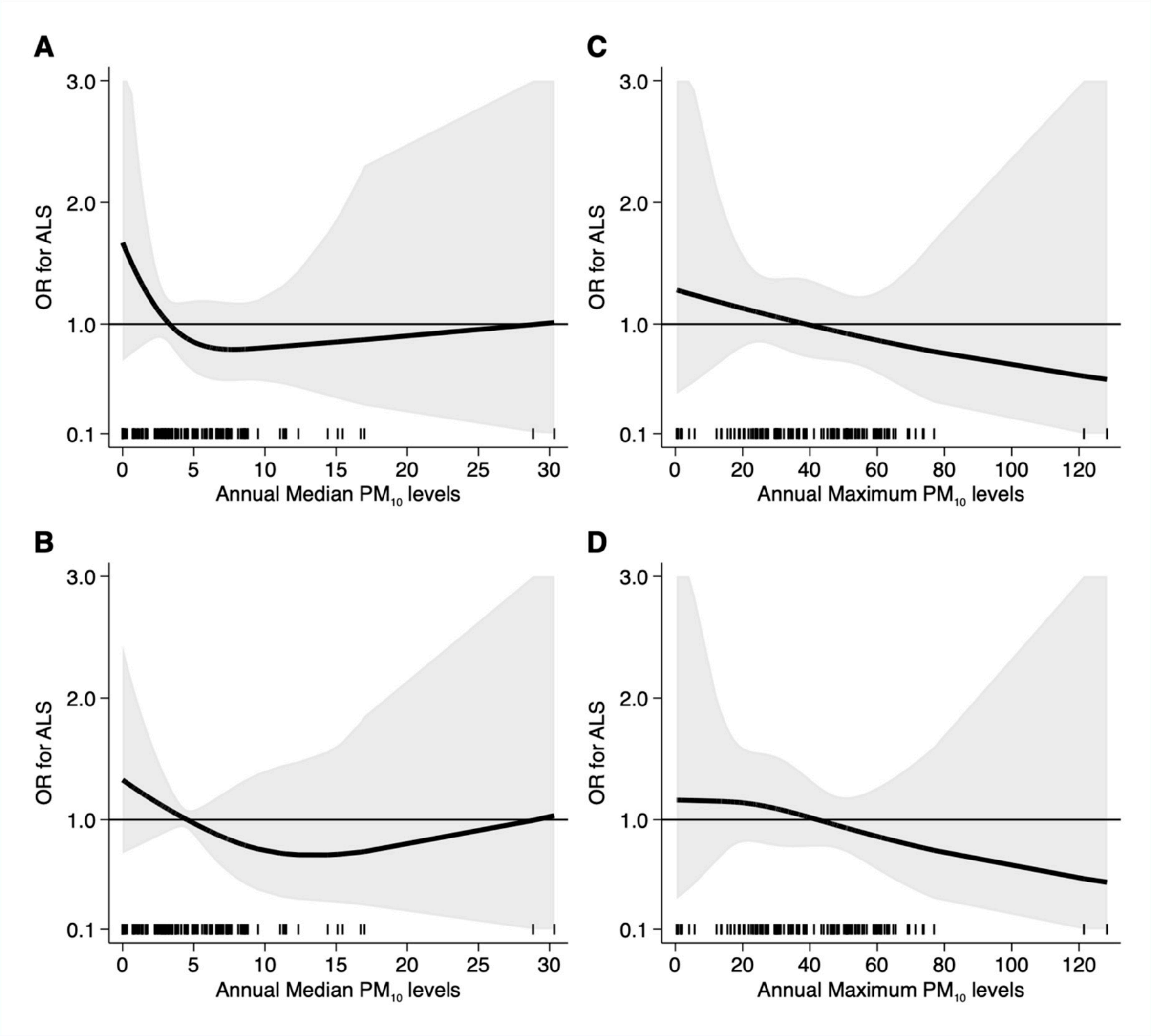

Figure S1. Spline regression analysis of amyotrophic lateral sclerosis (ALS) risk for increasing particulate matter (PM_{10}) exposure for both annual median and maximum levels using 10th, 50th, and 90th percentile distribution (A, C) or fixed categories (B: 5, 10, and 20 μg/m³; D: 10, 20, and 50 μg/m³) as cutpoints with further adjustment for agricultural land use. The black line indicates the odds ratio for ALS risk; the dash gray area indicates 95% confidence limits; the reference line is at 1.0; black spikes indicate the distribution of participant PM_{10} levels.

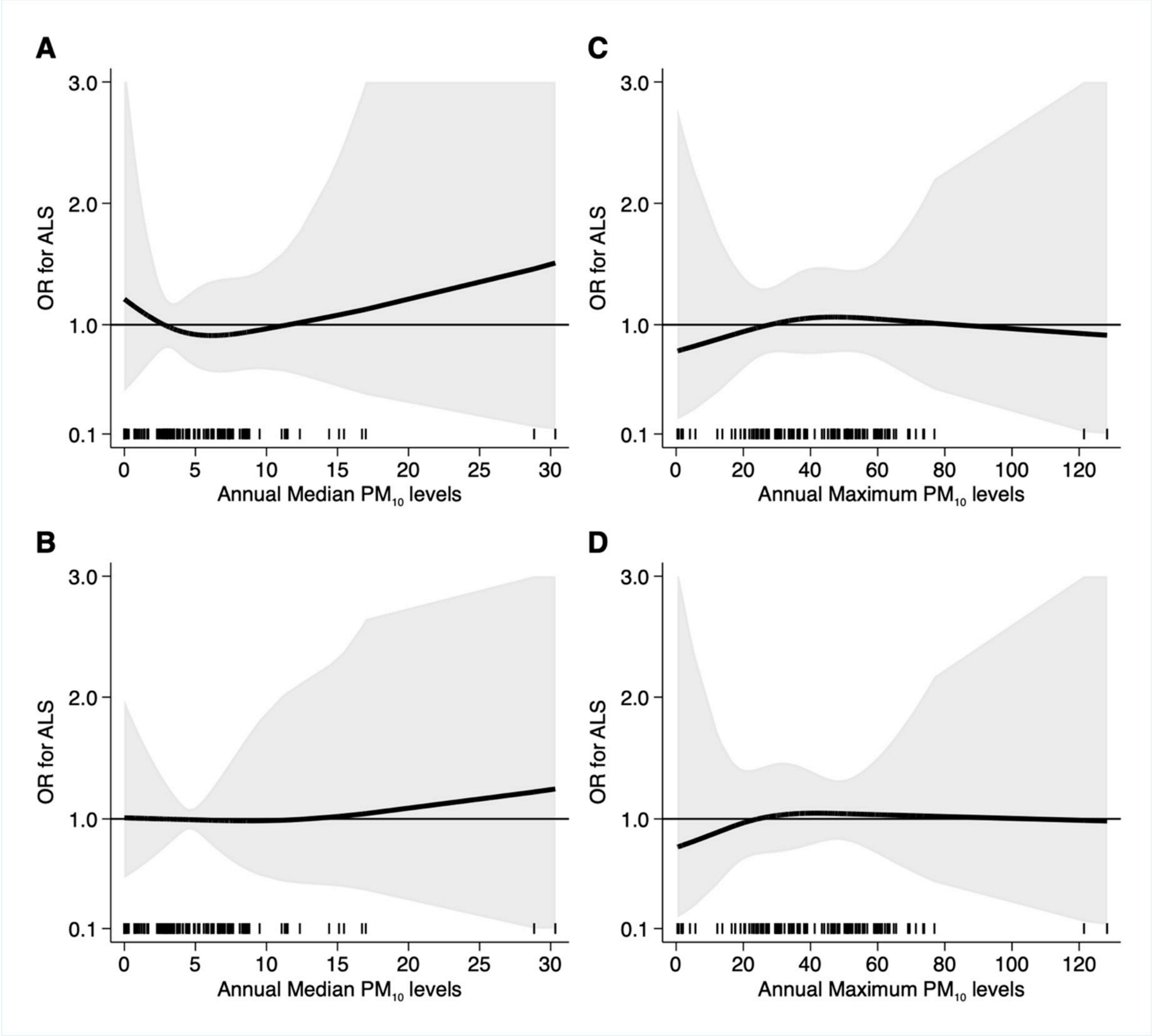

Figure S2. Spline regression analysis of amyotrophic lateral sclerosis (ALS) risk for increasing particulate matter (PM_{10}) exposure for both annual median and maximum levels using 10th, 50th, and 90th percentile distribution (A, C) or fixed categories (B: 5, 10, and 20 μg/m³; D: 10, 20, and 50 μg/m³) as cutpoints with further adjustment for agricultural land use. The black line indicates the odds ratio for ALS risk; the dash gray area indicates 95% confidence limits; the reference line is at 1.0; black spikes indicate the distribution of participant PM_{10} levels.

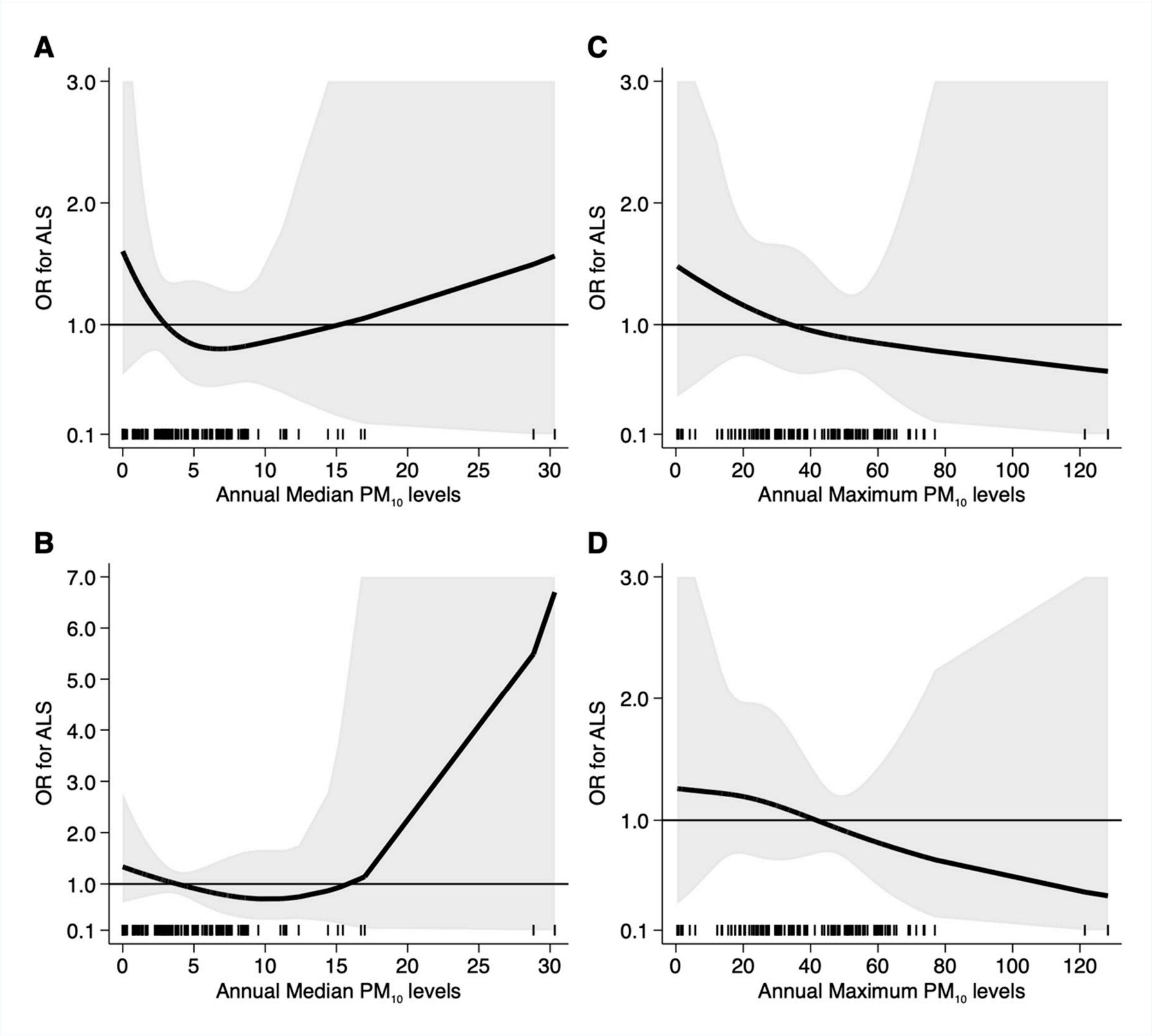

Figure S3. Spline regression analysis of amyotrophic lateral sclerosis (ALS) risk for increasing particulate matter (PM_{10}) exposure for both annual median and maximum levels using 10th, 50th, and 90th percentile distribution (A, C) or fixed categories (B: 5, 10, and 20 μg/m^3; D: 10, 20, and 50 μg/m^3) as cutpoints with further adjustment for inorganic selenium levels in cerebrospinal fluid. The black line indicates the odds ratio for ALS risk; the dash gray area indicates 95% confidence limits; the reference line is at 1.0; black spikes indicate the distribution of participant PM_{10} levels.

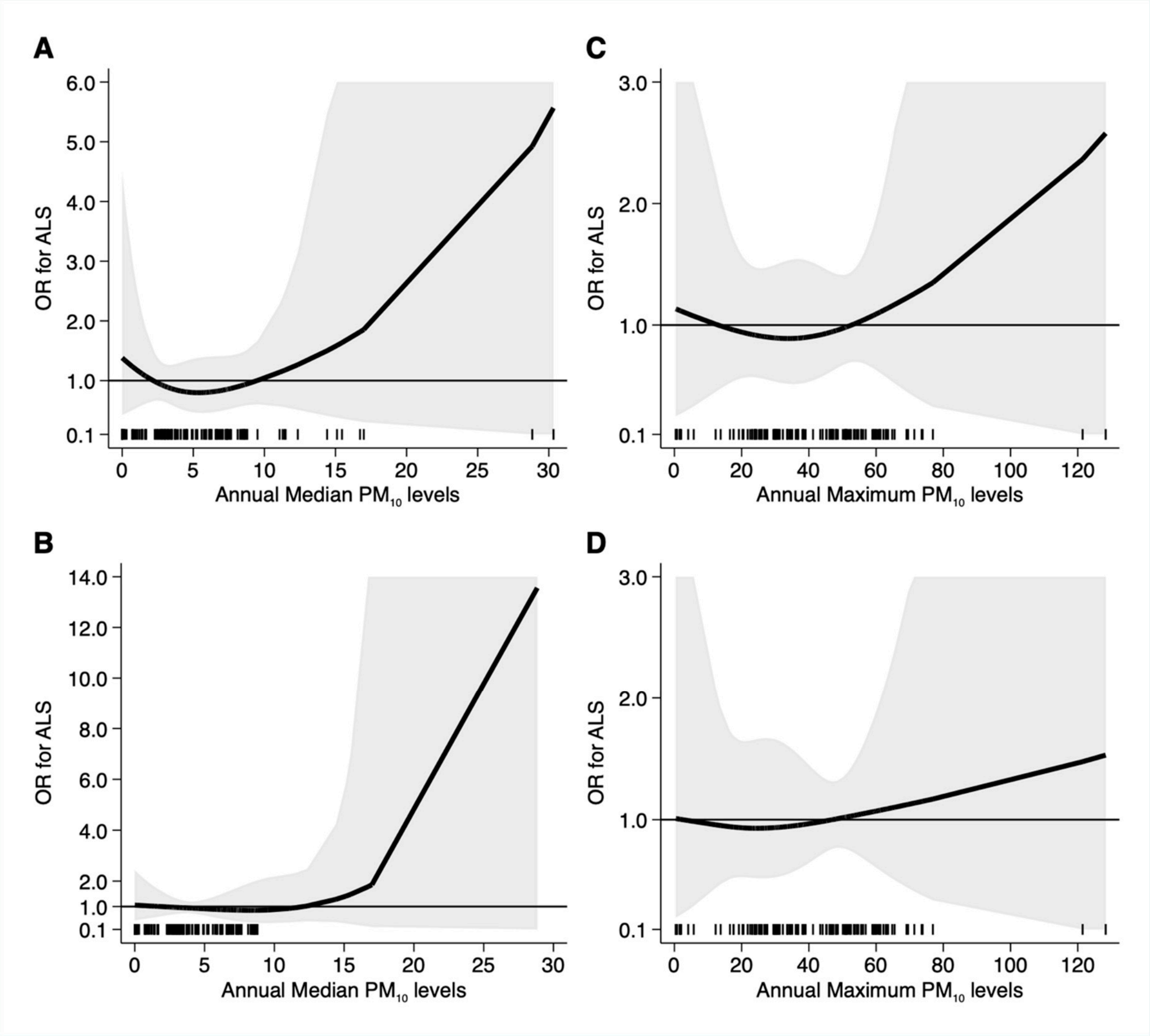

Figure S4. Spline regression analysis of amyotrophic lateral sclerosis (ALS) risk for increasing particulate matter (PM_{10}) exposure for both annual median and maximum levels using 10th, 50th, and 90th percentile distribution (A, C) or fixed categories (B: 5, 10, and 20 μg/m^3; D: 10, 20, and 50 μg/m^3) as cutpoints with further adjustment for inorganic selenium levels in cerebrospinal fluid. The black line indicates the odds ratio for ALS risk; the dash gray area indicates 95% confidence limits; the reference line is at 1.0; black spikes indicate the distribution of participant PM_{10} levels.
